# Sleep Macrostructure and NREM Sleep Instability Analysis in Pediatric Developmental Coordination Disorder

**DOI:** 10.3390/ijerph16193716

**Published:** 2019-10-02

**Authors:** Maria Esposito, Francesco Precenzano, Ilaria Bitetti, Ilaria Zeno, Eugenio Merolla, Maria Cristina Risoleo, Valentina Lanzara, Marco Carotenuto

**Affiliations:** Clinic of Child and Adolescent Neuropsychiatry, Department of Mental Health, Physical and Preventive Medicine, Università degli Studi della Campania “Luigi Vanvitelli,” via Sergio Pansini 5, 80100 Naples, Italy; f.precenzano@hotmail.it (F.P.); ilaria.bitetti@gmail.com (I.B.); ilaria.zeno@gmail.com (I.Z.); eug.merolla@gmail.com (E.M.); mariacristinarisoleo@yahoo.it (M.C.R.); valelanz87@hotmail.it (V.L.); marco.carotenuto@unicampania.it (M.C.)

**Keywords:** developmental coordination disorder, children, sleep, CAP, PSG

## Abstract

Developmental Coordination Disorder (DCD) is considered to be abnormal motor skills learning, identified by clumsiness, slowness, and/or motor inaccuracy impairing the daily-life activities in all ages of life, in the absence of sensory, cognitive, or neurological deficits impairment. The present research focuses on studying DCD sleep structure and Cyclic Alternating Pattern (CAP) parameters with a full overnight polysomnography and to study the putative correlations between sleep architecture and CAP parameters with motor coordination skills. The study was a cross-sectional design involving 42 children (26M/16F; mean age 10.12 ± 1.98) selected as a DCD group compared with 79 children (49M/30F; mean age 9.94 ± 2.84) identified as typical (no-DCD) for motor ability and sleep macrostructural parameters according to the MABC-2 and polysomnographic (PSG) evaluations. The two groups (DCD and non-DCD) were similar for age (*p* = 0.715) and gender (*p* = 0.854). More significant differences in sleep architecture and CAP parameters were found between two groups and significant correlations were identified between sleep parameters and motor coordination skills in the study population. In conclusion, our data show relevant abnormalities in sleep structure of DCD children and suggest a role for rapid components of A phases on motor coordination development

## 1. Introduction

Motor coordination is a generic term to identify ability in planning and performing motor skills according to the biological principles of efficiency and economy.

In general, many clinical conditions may impact motor skills such as epileptic syndromes, autism spectrum disorders, Attention Deficit Hyperactivity Disorder (ADHD), primary headaches, learning disorders, and obesity [1,2], whereas, when motor coordination is primarily impaired, Developmental Coordination Disorder (DCD) may be identified.

According to the American Psychiatric Association (APA) criteria [3], DCD is considered as abnormal motor skills learning identified by clumsiness, slowness, and/or motor inaccuracy impairing the daily-life activities in all age of life, in the absence of sensory, cognitive, or neurological deficits impairment [3]. In this picture, DCD can impair many relevant adaptive domains such as self-care activities, education levels, self-employment, self-efficacy, and self-esteem.

In 2011 Barnett et al. firstly suggested the putative link of DCD with sleep disorders based on a questionnaire-report study pinpointing the higher prevalence of bedtime resistance, NREM parasomnias, and daytime sleepiness in DCD school-children, suggesting that sleep patterns of children with DCD may be of clinical relevance and are worthy of further investigation [4]. From the same perspective, in 2016 Wiggs et al., with an actigraphic study on DCD children, confirmed the co-occurrence of sleep troubles and DCD in a small group of affected school-children that showed a significant reduction in sleep duration parameters, with respect to healthy controls, with the consequence of daytime fatigue, pre-sleep arousal, and daytime sleepiness [5].

On the other hand, it is well known that sleep troubles could impact significantly on many daily life activities [6,7,8], and in many learning processes at all ages of life [9,10]

In this point of view some studies pinpoint the role of sleep in motor learning tasks and on motor memory consolidation [11,12], showing a link between specific sleep features (sleep spindles and REM sleep) and sleep dependent motor memory consolidation [10,13]

Considering that nowadays to the best of our knowledge there are no specific polysomnographic studies among children affected by DCD, the present study design has been focused on the following topics:

1. to study the putative correlations between sleep macrostructure and NREM instability and motor coordination skills;
2. to study DCD sleep structure and NREM instability with a full overnight polysomnography in order to compare the data with the sleep structure of a group of unaffected children.

## 2. Materials and Methods

### 2.1. Ethical Approval Statement

Parents of children belonging to both groups provided written informed consent and a parent or guardian of any child participant provided written informed consent on their behalf.

The study design was approved by Departmental Ethics Committee and was carried out according to the Declaration of Helsinki criteria [14] (Prot. N. 13883; n. 2015-001161-36).

### 2.2. Study Design

The study was a cross-sectional design involving 42 children (26 males, 16 females; mean age 10.12 ± 1.98) selected as the Developmental Coordination Disorder (DCD) group, according to DSM-5 criteria from a starting population composed by 146 children (89 males, mean age 10.32 ± 2.05 years) consecutively referred to the Clinic of Child and Adolescent Neuropsychiatry at Università degli Studi della Campania “Luigi Vanvitelli” between February 2011 and June 2013 (Figure S1). Moreover, 79 children (49 males, 30 females; mean age 9.94 ± 2.84) were identified as typical (non-DCD) for motor ability and sleep macrostructural parameters according to the MABC-2 and polysomnographic (PSG) evaluations. (Prot. N. 13883; n. 2015-001161-36).

Moreover, 79 children (49 males, 30 females; mean age 9.94 ± 2.84) were identified as typical (non-DCD) for motor ability and sleep macrostructural parameters according to the MABC-2 and polysomnographic (PSG) evaluations. (Prot. N. 13883; n. 2015-001161-36).

Children of both groups (DCD and non-DCD) underwent an overnight full polysomnographic study (PSG) and a motor-coordination, visual–motor integration evaluation

All subjects were recruited from the same urban area, were all Caucasian, and had middle-class socioeconomic status.

### 2.3. Population Study

The whole study population was composed by 121 children (75 males, mean aged 10.03 ± 2.76 years) consecutively referred to the Clinic of Child and Adolescent Neuropsychiatry at Università degli Studi della Campania “Luigi Vanvitelli” between February 2011 and June 2013. In order to select the DCD children group, all children underwent a Neurological and psychological examination (including M-ABC and VMI tools) to identify the presence of DSM-5 diagnostic criteria. From this perspective, for all children we have provided a cognitive evaluation (using Wechsler Intelligence scales) and we have excluded all children with a cognitive impairment (IQ < 2SD). Moreover, the following other exclusion criteria were chosen: genetic disease (i.e., Down syndrome, Fragile-X syndrome, Prader-Willi syndrome), epilepsy, cerebral palsy, intellectual disability (IQ < 70), obesity, autism spectrum disorder, psychiatric disorder, born prematurely (gestational age < 36 weeks), born with very low birth weight (<1500 g).

### 2.4. Polysomnographic Evaluation (PSG)

According to the current international criteria [15,16], full overnight PSG recordings were performed for all studied children starting at the children’s usual bedtime and went on until spontaneous morning awakening.

All PSG recordings were visually scored using the Hypnolab 1.2 sleep software analysis (SWS Soft, Italy) and the conventional sleep parameters (Time in bed-TIB; Sleep period time-SPT; Total sleep time-TST; Sleep latency-SL; First REM latency-FRL; Number of stage shifts/hour-SS/h; Number of awakenings/hour-AWN/h; Sleep efficiency-SE%; Percentage of SPT in wakefulness after sleep onset-WASO%; Percentage of SPT in sleep stages 1-N1%, 2-N2%, slow-wave sleep-N3%, and REM sleep-REM%) were identified. Moreover, nocturnal respiratory events were scored according to the international criteria for pediatric age, considering the apnea–hypopnea index (AHI) as the number of apneas and hypopneas per hour of total sleep time; pathological if > 1.2–1.3 [17]. Sleep periodic limb movements (PLMs) events were analyzed [18] and a PLMI ≥ 5 was considered pathological.

### 2.5. Cyclic Alternating Pattern Analysis

The NREM sleep instability was studied using the cyclic alternating pattern (CAP) evaluation according to the standard criteria coded by Terzano et al. [19]. In this perspective, in order to perform CAP analysis of studied children, we evaluated the following CAP parameters: CAP time in NREM sleep; CAP rate%; number and duration of CAP cycles; number and duration of CAP sequences; number, duration, and percentage of A phases (including the phase A subtypes); A1 index; A2 index; A3 index; number and duration of B phases.

### 2.6. Motor-Coordination Assessment

The motor coordination performance of children of both groups was evaluated with the Movement Assessment Battery for Children (M-ABC) [20]. This tool was widely applied during the recruitment period of our sample in both clinical and research settings to support the DCD diagnosis. M-ABC required about 20–40 min/children to assess the fine and gross motor skills using three manual dexterity tasks, two ball skills tasks, and three balance tasks. In the present study DCD was diagnosed, according to DSM-5 criteria, when the M-ABC total score was ≤ 5 percentile, and a borderline motor impairment when the total score was ≤ 15 percentile.

### 2.7. Visual-Motor Integration Evaluation

The Beery visual-motor integration (VMI) [21] task is a tool designed for children with administration time of about 10 min/child. VMI was used to assess fine motor coordination and visual motor integration among children of both groups. The Beery VMI test is a paper-and-pencil test that require to children to copy up to 27 geometric forms with increasing complexity. The percentile scores were used for diagnosing the visual–motor abnormalities in our sample, and a value ≤ 5 percentile was considered to mean a visual–motor integration impairment.

### 2.8. Statistical Analysis

The Chi-square and the *t*-Test analysis were used when appropriate in order to verify the groups similarity for age and gender.

In order to analyze the relationship among variables of sleep and CAP with M-ABC and VMI variables, the Pearson’s correlation test was computed.

The comparisons between sleep architecture and CAP parameters obtained in DCD and Control groups were carried out by the Mann–Whitney U test, considering the relative small size of recruited population.

*p* value < 0.05 was considered statistically significant.

All data were coded and analyzed using the commercially available STATISTICA 6.0 package for Windows (StatSoft, Inc., Tulsa, OK, USA).

## 3. Results

The two groups (DCD and non-DCD) were similar for age (*p* = 0.715) and gender (*p* = 0.854).

Based on macrostructural sleep parameters, DCD children showed a significant reduction in all sleep duration parameters than the control group (TIB *p* = 0.003; SPT *p* = 0.003; TST *p* = 0.001) and in REM% (*p* < 0.001) (Table 1), while no significant differences were found in sleep respiratory and movement parameters (Table 1).

According to CAP analysis, DCD children showed a higher quote of S1-CAP rate% (30.693 ± 29.634 vs. 13.818 ± 16.688; *p* < 0.001), and Tot num A2% (31.750 ± 23.879 vs. 19.266 ± 23.080; *p* = 0.006), Tot num A3% (15.771 ± 8.272 vs. 6.066 ± 2.465; *p* < 0.001), A3 index (6.669 ± 5.203 vs. 2.425 ± 1.796; *p* < 0.001), A mean duration (11.412 ± 5.931 vs. 8.232 ± 4.525; *p* = 0.001), A1 mean duration (9.488 ± 5.657 vs. 6.804 ± 3.870; *p* = 0.003), A2 mean duration (12.690 ± 6.883 vs. 10.224 ± 4.975; *p* = 0.025) than control group (Table 2).

Moreover, DCD children showed a significant reduction in SWS-CAP rate% (43.64 ± 16.584 vs. 49.443 ± 10.591; *p* = 0.021), Tot num A1% (52.479 ± 25.479 vs. 74.673 ± 22.477; *p* < 0.001), A1 index (26.231 ± 19.588 vs. 36.515 ± 13.206; *p* = 0.001), B mean duration (19.643 ± 4.397 vs. 23.095 ± 3.753; *p* < 0.001) (Table 2).

Table 3 shows the Pearson’s analysis correlation among M-ABC, VMI, and PSG parameters pinpointing a statistical positive relationship between balance scores and SS/h (r = 0.189, *p* = 0.038), and Awk/h (r = 0.232, *p* = 0.010), between dexterity scores and N1% (r = 0.215, *p* = 0.018), between M-ABC global score and REM% (r = 0.221, *p* = 0.015), while a negative relationship has been identified between REM % and ball interaction skill (r = −0.214, *p* = 0.018) and balance (r = −0.265, *p* = 0.003), and between TST and ball interaction skills (r = −0.184, *p* = 0.043).

According to the CAP analysis significant relationships were found between M-ABC and VMI scores and A phase components (A1, A2, and A3) (Table 4).

Table 1 shows comparison of PSG sleep parameters (Time in bed-TIB; Sleep period time-SPT; Total sleep time-TST; Sleep latency-SL; First REM latency-FRL; Number of stage shifts/hour-SS/h; Number of awakenings/hour-AWN/h; Sleep efficiency-SE%; Percentage of SPT in wakefulness after sleep onset-WASO%; Percentage of SPT in sleep stages 1-N1%, 2-N2%, slow-wave sleep-N3%, and REM sleep-REM%) between DCD and Non-DCD children. 

The Mann–Whitney U test was applied *p* value < 0.05 was considered statistically significant.

Table 2 shows comparison of CAP parameters (CAP time in NREM sleep; CAP rate%; number and duration of CAP cycles; number and duration of CAP sequences; number, duration, and percentage of A phases (including the phase A subtypes); A1 index; A2 index; A3 index; number and duration of B phases) between DCD and Non-DCD children.

The Mann–Whitney U test was applied *p* value < 0.05 was considered statistically significant.

Table 3 shows correlation analysis between M-ABC, VMI scores, and PSG sleep parameters (Time in bed-TIB; Sleep period time-SPT; Total sleep time-TST; Sleep latency-SL; First REM latency-FRL; Number of stage shifts/hour-SS/h; Number of awakenings/hour-AWN/h; Sleep efficiency-SE%; Percentage of SPT in wakefulness after sleep onset-WASO%; Percentage of SPT in sleep stages 1-N1%, 2-N2%, slow-wave sleep-N3%, and REM sleep-REM%).

Pearson correlation analysis was applied *p* values < 0.05 were considered significant.

Table 4 shows correlation analysis between M-ABC, VMI scores, and CAP parameters (CAP time in NREM sleep; CAP rate%; number and duration of CAP cycles; number and duration of CAP sequences; number, duration, and percentage of A phases (including the phase A subtypes); A1 index; A2 index; A3 index; number and duration of B phases).

Pearson correlation analysis was applied; *p* values < 0.05 were considered significant.

## 4. Discussion

The findings of the present study highlighted firstly both differences in the sleep macrostructural parameters and the relationship between the NREM sleep instability and motor coordination ability in children affected by DCD, with respect to a sample of typically developing children.

There are more studies about the role of sleep in motor ability and motor learning [12,22] at any age of life [22,23]. Particularly, an interesting report of Bothe et al. [22] in 2018 showed the impact of sleep on gross motor adaptation in a group of adolescents with typical developing profile, pinpointing the role of an increase in REM sleep on cortico-cerebellar network activity that organizes dynamic aspects of movement such as motor coordination. From this perspective, we can explain the REM% reduction in our sample as a sign of the alteration in cortico-cerebellar network activity that could be considered as a cause of DCD [24].

In 2012 Barnett et al. [4] has been explored the possible relationship between DCD and sleep, highlighting that subscale scores indicated particular problems with bedtime resistance, parasomnias and daytime sleepiness, and no differences between the groups for sleep onset delay, sleep duration, night wakings, and sleep-disordered breathing, suggesting that sleep patterns of children with DCD may be of clinical relevance. In general, the close relationship between sleep and motor skills is well known [25], while to date no PSG reports were published to pinpoint the putative link between sleep macrostructural fragmentation and motor ability. In this picture, the present study highlights the relationship between SS/h and AWK/h identifiable as a proof of sleep disruption and balance ability, as reported in adult subjects [25]. About the positive correlation between REM% and general motor skills, these data may be interpreted according to the clear role of REM sleep in verbal and non-verbal learning [26,27,28,29].

The present study may be considered the first based on polysomnographic data performed on children affected by DCD. Moreover, firstly the analysis of Cyclic Alternating Pattern during the NREM sleep was performed as a putative explanation of DCD as showed by our data of positive correlation between motor skills and rapid CAP A2 and A3.

It is well known that in dynamic organization of sleep CAP could express a condition of instability of the vigilance level that translates the brain effort to preserve and regulate sleep macrostructure.

Specific alterations of phase A subtypes have been described in a number of sleep disorders such as nocturnal frontal lobe epilepsy [30], sleep apnea [31], insomnia [32,33], and narcolepsy [34]

In particular, subtypes A1 are mostly involved in the build-up and consolidation of slow-wave sleep, while subtypes A2 and A3 are closely related and modulate the onset of REM sleep [35,36].

A1 alterations are present in many developmental disorders with intellectual disabilities (i.e., PWS, fragile X, Down) according to the specific location of the generator of A1 in the prefrontal cortex, that explain the close relationship between A1 activity and intellectual level or more generally with the knowledge and creative processes.

Conversely, even if the role of rapid components of A phases (A2 and A3) is not completely understood, it is known that A3 generators are probably allocated in the pyramidal cortex suggesting a possible relationship with ancestral aspects such as adjustment of the movement. The link between CAP regulation and motor activity is also substantiated by the effects of physical stimulation (massage) on sleep maturation [37,38] and by the effects of physical fitness on sleep efficiency at any age [39,40,41].

Accordingly, subtypes A1 dominate in the first part of the sleep cycle where they accompany the progressive transition from light sleep (stages 1 and 2) to deep sleep (stages 3 and 4) and therefore appear involved in the process of build-up and maintenance of EEG synchronization [42,43,44]. By contrast, subtypes A2 and A3 prevail physiologically in the final part of the sleep cycle, where they disrupt EEG synchronization and prepare the appropriate desynchronized background for the onset of REM sleep [42,43,44].

About the topographic localization, the subtypes A1 are essentially expressions of transient activation restricted to the frontal lobe which seldom crosses the fronto-occipital midline; on the contrary, subtypes A3 are projected into the parieto-occipital regions and subtypes A2, with mixed slow-rapid components, span from frontal to occipital lobes [45]. Considering the role of the parietal lobe, also in proprioception and somatosensation, our findings in the alterations of subtypes A2 and A3 over-expression could be interpreted as a sort of compensative mechanism for equilibrium maintainance in NREM sleep instability balancing.

Interestingly, A2 and A3 CAP parameters seem to be closely related to motor coordination ability, even if no clear explanation may be provided about it except that A3 CAP generators seem to be allocated near the main motor cortex area [45].

On the other hand, we have to consider as the main limitations of the present study the relatively small size of the recruited population (although the DCD diagnosis was carried out according to the international criteria excluding many other pathological conditions) and the not complete consideration of confounding factors on DCD-sleep link evaluation.

Notwithstending these limitations, the strength of our study may be identified in the use of the gold standard neurophysiological tool for sleep study such as the polysomnographic evaluation that comprised not only the classical macrostructural sleep study, but mainly the NREM sleep instability analysis not routinely performed in developmental age.

In conclusion, our PSG study showed firstly the presence of sleep disturbances in DCD children that could be considered a relevant comorbidity in this population, confirming findings of previous questionnaires and actigraphical-based studies [4,5]. Moreover, our results highlight a possible etiological link between NREM sleep instability organization and motor coordination disorders in DCD children, suggesting a relevant role for rapid component of A phases (A2 and A3) on motor coordination development.

Finally, further studies are need to confirm our findings in a larger DCD population and to improve the clinical management of these children

## Figures and Tables

**Table 1:** Comparison of polysomnographic (PSG) sleep architecture between Developmental Coordinaton Disorder (DCD) and Non-DCD children.

	DCD (N = 42)	No−DCD (N = 79)	U	Z	*p*
**TIB-min**	**509.637 ± 62.421**	**554.652 ± 84.406**	**1029.000**	**3.430**	**0.001**
**SPT-min**	**481.583 ± 63.010**	**525.253 ± 79.992**	**1015.000**	**3.506**	**<0.001**
**TST-min**	**443.006 ± 79.506**	**495.918 ± 84.913**	**963.000**	**3.789**	**<0.001**
**SOL-min**	18.994 ± 14.528	18.734 ± 15.928	1655.000	−0.022	0.983
**FRL-min**	140.446 ± 60.754	129.753 ± 57.100	1498.500	−0.874	0.382
**SS-h**	8.799 ± 3.454	8.554 ± 3.248	1624.000	−0.191	0.849
**AWN-h**	2.954 ± 2.401	2.273 ± 1.935	1408.000	−1.367	0.172
**SE%**	86.820 ± 10.448	89.322 ± 7.351	1438.000	1.203	0.229
**WASO-spt**	8.232 ± 9.749	5.738 ± 6.652	1418.500	−1.309	0.190
**S1-spt**	2.795 ± 4.063	2.09 ± 2.453	1641.500	−0.095	0.924
**S2-spt**	40.931± 9.608	40.977± 20.535	1376.000	−1.541	0.123
**SWS-spt**	32.054 ±12.203	32.587± 9.239	1560.000	0.539	0.590
**REM-spt**	**15.975 ± 7.781**	**20.825 ± 6.598**	**1092.000**	**3.087**	**0.002**

**Table 2:** Comparison of Cyclic Alternating Pattern (CAP) parameters between DCD and Non-DCD children.

	DCD (n = 42)	No-DCD (N = 79)	U	Z	*p*
**CAP_tot_num**	**319.000 ± 127.098**	**357.734 ± 91.494**	**1277.500**	**2.077**	**0.038**
**CAP_Rate%**	**31.671 ± 13.221**	**35.222 ± 9.225**	**1223.000**	**2.374**	**0.018**
**CAP_Rate%S1**	**30.693 ± 29.634**	**13.818 ± 16.688**	**1226.500**	**−2.083**	**0.037**
**CAP_Rate%S2**	**22.774 ± 14.178**	**26.614 ± 11.689**	**1283.000**	**2.047**	**0.041**
**CAP_Rate%SWS**	**43.640 ± 16.584**	**49.443 ± 10.591**	**1168.000**	**2.673**	**0.008**
**Tot_num_A1%**	**52.479 ± 25.479**	**74.673 ± 22.477**	**630.000**	**5.603**	**<0.001**
**Tot_num_A2%**	**31.750 ± 23.879**	**19.266 ± 23.080**	**998.000**	**−3.599**	**<0.001**
**Tot_num_A3%**	**15.771 ± 8.272**	**6.066 ± 2.465**	**3.000**	**−9.016**	**<0.001**
**A_mean_dur**	**11.412 ± 5.931**	**8.232 ± 4.525**	**1101.000**	**−3.038**	**0.002**
**A1_mean_dur**	**9.488 ± 5.657**	**6.804 ± 3.870**	**1278.000**	**−2.074**	**0.038**
**A2_mean_dur**	12.690 ± 6.883	10.224 ± 4.975	1439.000	−1.198	0.231
**A3_mean_dur**	14.186 ± 5.096	15.482 ± 7.560	1519.500	0.759	0.448
**A1_index**	**26.231 ± 19.588**	**36.515 ± 13.206**	**1117.000**	**2.951**	**0.003**
**A2_index**	**12.740 ± 10.976**	**9.037± 10.884**	**1175.000**	**−2.635**	**0.008**
**A3_index**	**6.669 ± 5.203**	**2.425 ± 1.796**	**583.500**	**−5.856**	**<0.001**
**B_mean_dur**	**19.643 ± 4.397**	**23.095 ± 3.753**	**947.000**	**3.877**	**<0.001**
**Cycle_mean_dur**	31.167 ± 7.130	31.225 ± 5.970	1570.000	0.485	0.628
**Num_of_seq**	33.167 ± 16.174	35.000 ± 8.251	1367.000	1.589	0.112
**Cyc_seq**	8.697± 3.808	8.445 ± 2.244	1416.500	1.320	0.187

**Table 3:** Pearson correlation analysis between Movement Assessment Battery for Children (M-ABC), visual-motor integration (VMI) scores and PSG sleep architecture.

	Dexterity	Ball Skills	Equilibrium	M-ABC Total Score	VMI %	Visual %	Motor %
**TIB-min**	−0.058 *p* = 0.530	**−0.194** ***p*** ** = 0.033**	−0.107 *p* = 0.244	0.062 *p* = 0.499	0.145 *p* = 0.113	−0.053 *p* = 0.562	0.100 *p* = 0.276
**SPT-min**	−0.092 *p* = 0.317	**−0.178** ***p*** ** = 0.050**	−0.084 *p* = 0.358	0.067 *p* = 0.463	0.162 *p* = 0.075	−0.021 *p* = 0.819	0.121 *p* = 0.187
**TST-min**	−0.110 *p* = 0.229	**−0.184** ***p*** ** = 0.043**	−0.115 *p* = 0.209	0.092 *p* = 0.318	0.171 *p* = 0.062	−0.046 *p* = 0.619	0.116 *p* = 0.206
**SOL-min**	0.146 *p* = 0.111	−0.159 *p* = 0.082	−0.096 *p* = 0.296	0.005 *p* = 0.957	0.026 *p* = 0.774	−0.067 *p* = 0.466	0.009 *p* = 0.926
**FRL-min**	0.006 *p* = 0.945	0.112 *p* = 0.220	0.018 *p* = 0.849	−0.009 *p* = 0.927	0.026 *p* = 0.780	−0.070 *p* = 0.444	0.033 *p* = 0.719
**SS-h**	−0.067 *p* = 0.467	0.028 *p* = 0.764	**0.189** ***p*** ** = 0.038**	−0.020 *p* = 0.830	−0.001 *p* = 0.991	0.112 *p* = 0.221	−0.017 *p* = 0.850
**AWN-h**	−0.009 *p* = 0.925	0.115 *p* = 0.210	**0.232** ***p*** ** = 0.010**	−0.092 *p* = 0.315	−0.023 *p* = 0.805	0.173 *p* = 0.058	−0.065 *p* = 0.478
**SE%**	−0.088 *p* = 0.339	−0.073 *p* = 0.425	−0.056 *p* = 0.544	0.066 *p* = 0.474	0.090 *p* = 0.326	−0.013 *p* = 0.892	0.047 *p* = 0.613
**WASO-spt**	0.063 *p* = 0.492	0.098 *p* = 0.285	0.104 *p* = 0.257	−0.080 *p* = 0.386	−0.071 *p* = 0.442	0.069 *p* = 0.449	−0.021 *p* = 0.821
**S1-spt**	**0.215** ***p*** ** = 0.018**	−0.075 *p* = 0.414	0.048 *p* = 0.601	−0.162 *p* = 0.075	−0.034 *p* = 0.710	−0.079 *p* = 0.391	−0.167 *p* = 0.068
**S2-spt**	0.093 *p* = 0.310	−0.004 *p* = 0.966	0.019 *p* = 0.834	−0.105 *p* = 0.251	0.015 *p* = 0.874	−0.029 *p* = 0.755	−0.014 *p* = 0.875
**SWS-spt**	−0.061 *p* = 0.504	0.113 *p* = 0.216	−0.023 *p* = 0.799	0.057 *p* = 0.534	0.018 *p* = 0.841	−0.049 *p* = 0.593	−0.032 *p* = 0.729
**REM-spt**	−0.146 *p* = 0.109	**−0.214** ***p*** ** = 0.018**	**−0.265** ***p*** ** = 0.003**	**0.221** ***p*** ** = 0.015**	0.126 *p* = 0.167	−0.066 *p* = 0.474	0.137 *p* = 0.133

**Table 4:** Pearson correlation analysis between M-ABC, VMI scores, and sleep CAP parameters.

	Dexterity	Ball Skills	Equilibrium	M-ABC Total Score	VMI %	Visual %	Motor %
**CAP_tot_num**	0.111 *p* = 0.226	−0.155 *p* = 0.090	−0.121 *p* = 0.188	−0.041 *p* = 0.653	0.089 *p* = 0.332	0.152 *p* = 0.096	−0.025 *p* = 0.782
**CAP_Rate%**	−0.001 *p* = 0.990	−0.153 *p* = 0.093	**−0.222** ***p*** ** = 0.014**	0.125 *p* = 0.172	0.081 *p* = 0.378	0.105 *p* = 0.252	−0.023 *p* = 0.799
**CAP_Rate%S2**	0.060 *p* = 0.517	−0.108 *p* = 0.239	−0.088 *p* = 0.336	−0.031 *p* = 0.738	0.057 *p* = 0.537	0.137 *p* = 0.133	−0.055 *p* = 0.547
**CAP_Rate%SWS**	−0.089 *p* = 0.333	**−0.192** ***p*** ** = 0.035**	**−0.226** ***p*** ** = 0.013**	**0.210** ***p*** ** = 0.021**	0.110 *p* = 0.232	0.091 *p* = 0.320	0.009 *p* = 0.923
**Tot_num_A1%**	**−0.191** ***p*** ** = 0.036**	**−0.185** ***p*** ** = 0.043**	**−0.339** ***p*** ** = 0.000**	**0.236** ***p*** ** = 0.009**	**0.351** ***p*** ** = 0.000**	0.013 *p* = 0.889	**0.220** ***p*** ** = 0.015**
**Tot_num_A2%**	0.020 *p* = 0.828	0.085 *p* = 0.355	**0.188** ***p*** ** = 0.039**	−0.042 *p* = 0.648	**−0.300** ***p*** ** = 0.001**	−0.025 *p* = 0.784	−0.134 *p* = 0.143
**Tot_num_A3%**	**0.635** ***p*** ** = 0.000**	**0.388** ***p*** ** = 0.000**	**0.602** ***p*** ** = 0.000**	**−0.725** ***p*** ** = 0.00**	**−0.260** ***p*** ** = 0.004**	0.040 *p* = 0.662	**−0.349** ***p*** ** = 0.000**
**A_mean_dur**	0.062 *p* = 0.498	**0.262** ***p*** ** = 0.004**	0.139 *p* = 0.127	−0.084 *p* = 0.363	**−0.254** ***p*** ** = 0.005**	−0.038 *p* = 0.676	−0.120 *p* = 0.191
**A1_mean_dur**	−0.004 *p* = 0.963	**0.286** ***p*** ** = 0.001**	0.119 *p* = 0.192	−0.046 *p* = 0.617	**−0.246** ***p*** ** = 0.007**	−0.061 *p* = 0.507	−0.073 *p* = 0.424
**A2_mean_dur**	0.035 *p* = 0.703	**0.229** ***p*** ** = 0.012**	0.094 *p* = 0.303	−0.067 *p* = 0.467	**−0.201** ***p*** ** = 0.027**	−0.061 *p* = 0.506	−0.058 *p* = 0.527
**A3_mean_dur**	0.076 *p* = 0.411	0.065 *p* = 0.477	−0.059 *p* = 0.524	−0.113 *p* = 0.216	0.026 *p* = 0.778	−0.043 *p* = 0.639	−0.071 *p* = 0.439
**A1_index**	−0.021 *p* = 0.818	**−0.179** ***p*** ** = 0.050**	**−0.254** ***p*** ** = 0.005**	0.074 *p* = 0.422	**0.259** ***p*** ** = 0.004**	0.090 *p* = 0.324	0.101 *p* = 0.271
**A2_index**	0.023 *p* = 0.799	0.002 *p* = 0.983	0.077 *p* = 0.405	0.007 *p* = 0.943	**−0.211** ***p*** ** = 0.020**	0.026 *p* = 0.774	−0.137 *p* = 0.135
**A3_index**	**0.579** ***p*** ** = 0.000**	**0.285** ***p*** ** = 0.002**	**0.399** ***p*** ** = 0.000**	**−0.589** ***p*** ** = 0.000**	−0.144 *p* = 0.115	0.080 *p* = 0.383	**−0.285** ***p*** ** = 0.002**
**B_mean_dur**	**−0.350** ***p*** ** = 0.000**	**−0.256** ***p*** ** = 0.005**	**−0.228** ***p*** ** = 0.012**	**0.387** ***p*** ** = 0.000**	**0.201** ***p*** ** = 0.027**	0.052 *p* = 0.574	**0.219** ***p*** ** = 0.016**
**Cycle_mean_dur**	**−0.184** ***p*** ** = 0.043**	0.049 *p* = 0.594	−0.028 *p* = 0.762	**0.190** ***p*** ** = 0.037**	−0.086 *p* = 0.348	0.004 *p* = 0.963	0.053 *p* = 0.567
**Num_of_seq**	0.082 *p* = 0.371	−0.118 *p* = 0.197	0.040 *p* = 0.660	−0.097 *p* = 0.292	0.054 *p* = 0.557	**0.221** ***p*** ** = 0.015**	−0.033 *p* = 0.721
**Cyc_seq**	0.159 *p* = 0.082	−0.024 *p* = 0.796	**−0.203** ***p*** ** = 0.025**	−0.020 *p* = 0.828	−0.001 *p* = 0.992	−0.162 *p* = 0.077	−0.015 *p* = 0.870

## Supplementary Materials

The following are available online at https://www.mdpi.com/1660-4601/16/19/3716/s1, Figure S1: Flow chart describes the study enrollment steps.
