# Supplementary figures and images for: Sleep Macrostructure and NREM Sleep Instability Analysis in Pediatric Developmental Coordination Disorder
Source: Int J Environ Res Public Health. 2019 Oct 2;16(19):3716. doi: 10.3390/ijerph16193716 (PMC6801607; doi:10.3390/ijerph16193716)

### eCONSORT Flow Diagram

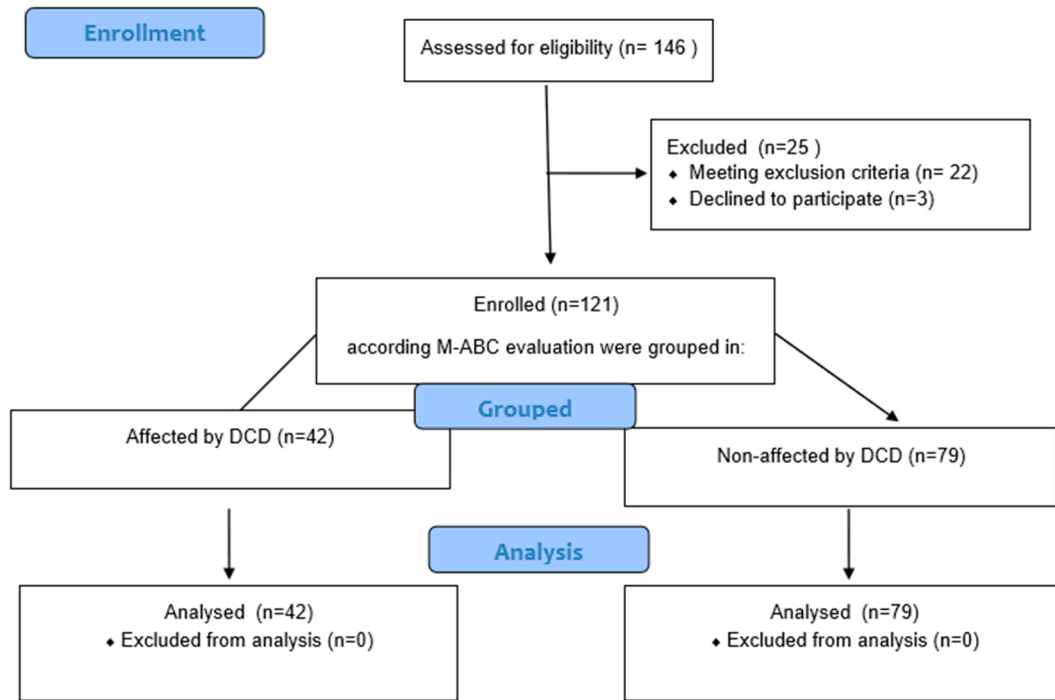

**Figure S1.** Flow chart describes the study enrollment steps.

Supplement: Supplementary file 1 [file ijerph-16-03716-s001.pdf]
